# The Deep Genome Project

**DOI:** 10.1186/s13059-020-1931-9

**Published:** 2020-02-03

**Authors:** K. C. Kent Lloyd, David J. Adams, Gareth Baynam, Arthur L. Beaudet, Fatima Bosch, Kym M. Boycott, Robert E. Braun, Mark Caulfield, Ronald Cohn, Mary E. Dickinson, Michael S. Dobbie, Ann M. Flenniken, Paul Flicek, Sanjeev Galande, Xiang Gao, Anne Grobler, Jason D. Heaney, Yann Herault, Martin Hrabě de Angelis, James R. Lupski, Stanislas Lyonnet, Ann-Marie Mallon, Fabio Mammano, Calum A. MacRae, Roderick McInnes, Colin McKerlie, Terrence F. Meehan, Stephen A. Murray, Lauryl M. J. Nutter, Yuichi Obata, Helen Parkinson, Michael S. Pepper, Radislav Sedlacek, Je Kyung Seong, Toshihiko Shiroishi, Damian Smedley, Glauco Tocchini-Valentini, David Valle, Chi-Kuang Leo Wang, Sara Wells, Jacqueline White, Wolfgang Wurst, Ying Xu, Steve D. M. Brown

**Affiliations:** 1 Department of Surgery, School of Medicine, and Mouse Biology Program, University of California, Davis, CA 95618 USA; 2 Wellcome Trust Sanger Institute, Hinxton, Cambridge, CB10 1SA UK; 3 Western Australian Register of Developmental Anomalies and Genetic Services of Western Australia, Department of Health, Government of Western Australia, Perth, Australia; 4 Division of Paediatrics and Telethon Kids Institute, Faculty of Health and Medical Sciences, University of Western Australia, Perth, Australia; 5 Faculty of Science and Engineering, School of Spatial Sciences, Curtin University, Perth, Australia; 6 Department of Molecular and Human Genetics, Baylor College of Medicine, Houston, TX 77030 USA; 7 Center of Animal Biotechnology and Gene Therapy, Universitat Autònoma Barcelona, Barcelona, Spain; 8 Children’s Hospital of Eastern Ontario Research Institute, University of Ottawa, Ottawa, ON K1H 8L1 Canada; 9 The Jackson Laboratory, Bar Harbor, ME 04609 USA; 10 Genomics England, William Harvey Research Institute, Queen Mary University of London, Charterhouse Square, London, EC1M 6BQ UK; 11 The Hospital for Sick Children, Toronto, ON M5G 1X8 Canada; 12 Departments of Molecular Physiology and Biophysics, Baylor College of Medicine, Houston, TX 77030 USA; 13 Phenomics Australia, The Australian National University, 131 Garran Road, Acton, ACT 2601 Australia; 14 The Centre for Phenogenomics, Lunenfeld-Tanenbaum Research Institute, Toronto, ON M5T 3H7 Canada; 15 European Molecular Biology Laboratory, European Bioinformatics Institute (EMBL-EBI), Wellcome Genome Campus, Hinxton, Cambridge, CB10 1SD UK; 16 National Facility for Gene Function in Health and Disease, Department of Biology, Indian Institute of Science, Education and Research (IISER) Pune, Pune, Maharashtra 411008 India; 17 SKL of Pharmaceutical Biotechnology and Model Animal Research Center, Collaborative Innovation Center for Genetics and Development, Nanjing Biomedical Research Institute, Nanjing University, Nanjing, 210061 China; 18 DST/NWU Preclinical Drug Development Platform, North-West University, Potchefstroom, 2520 South Africa; 19 Université de Strasbourg, CNRS, INSERM, Institut de Génétique, Biologie Moléculaire et Cellulaire, Institut Clinique de la Souris, IGBMC, PHENOMIN-ICS, 67404 Illkirch, France; 20 German Mouse Clinic, Institute of Experimental Genetics, Helmholtz Zentrum München, German Research Center for Environmental Health, 85764 Neuherberg, Germany; 21 Chair of Experimental Genetics, Center of Life and Food Sciences Weihenstephan, Technische Universität München, 85354 Freising-Weihenstephan, Germany; 22 German Center for Diabetes Research (DZD), 85764 Neuherberg, Germany; 23 Institut Imagine, UMR-1163 INSERM et Université de Paris, Hôpital Universitaire Necker-Enfants Malades, 24, Boulevard du Montparnasse, 75015 Paris, France; 24 Medical Research Council Harwell Institute (Mammalian Genetics Unit and Mary Lyon Centre), Harwell, Oxfordshire OX11 0RD UK; 25 Monterotondo Mouse Clinic, Italian National Research Council (CNR), Institute of Biochemistry and Cell Biology (IBBC), Monterotondo Scalo, I-00015 Rome, Italy; 26 Department of Medicine, Brigham and Women’s Hospital, Harvard Medical School, Boston, USA; 27 Lady Davis Research Institute, Jewish General Hospital, McGill University, 3999 Côte Ste- Catherine Road, Montreal, Quebec H3T 1E2 Canada; 28 The Centre for Phenogenomics, The Hospital for Sick Children, Toronto, ON M5T 3H7 Canada; 29 RIKEN BioResource Research Center, Tsukuba, Ibaraki, 305-0074 Japan; 30 Institute for Cellular and Molecular Medicine, Department Immunology, and SAMRC Extramural Unit for Stem Cell Research and Therapy, Faculty of Health Sciences, University of Pretoria, Pretoria, South Africa; 31 Czech Centre for Phenogenomics, Institute of Molecular Genetics of the Czech Academy of Sciences, 252 50 Vestec, Czech Republic; 32 Korea Mouse Phenotyping Consortium (KMPC) and BK21 Program for Veterinary Science, Research Institute for Veterinary Science, College of Veterinary Medicine, Seoul National University, 599 Gwanangno, Gwanak-gu, Seoul, 08826 South Korea; 33 Clinical Pharmacology, William Harvey Research Institute, School of Medicine and Dentistry, Queen Mary University of London, London, EC1M 6BQ UK; 34 McKusick-Nathans Department of Genetic Medicine, The Johns Hopkins University School of Medicine, 519 BRB, 733 N Broadway, Baltimore, MD 21205 USA; 35 National Laboratory Animal Center, National Applied Research Laboratories, Taipei, Taiwan; 36 Institute of Developmental Genetics, Helmholtz Zentrum München, German Research Center for Environmental Health GmbH, 85764 Neuherberg, Germany; 37 Chair of Developmental Genetics, Center of Life and Food Sciences Weihenstephan, Technische Universität München, 85354 Freising-Weihenstephan, Germany; 38 Deutsches Zentrum für Neurodegenerative Erkrankungen (DZNE), Munich Cluster for Systems Neurology (SyNergy), Adolf-Butenandt-Institut, Ludwig Maximillian’s Universitat Munchen, 81377 Munich, Germany; 39 Cambridge-Suda Genomic Resource Center, Jiangsu Key Laboratory of Neuropsychiatric Diseases, Medical College of Soochow University, Suzhou, 215123 Jiangsu China

## Introduction

In vivo research is critical to the functional dissection of multi-organ systems and whole organism physiology, and the laboratory mouse remains a quintessential animal model for studying mammalian, especially human, pathobiology. Enabled by technological innovations in genome sequencing, mutagenesis and genome editing, phenotype analyses, and bioinformatics, in vivo analysis of gene function and dysfunction in the mouse has delivered new understanding of the mechanisms of disease and accelerated medical advances. However, many significant hurdles have limited the elucidation of mechanisms underlying both rare and complex, multifactorial diseases, leaving significant gaps in our scientific knowledge. Future progress in developing a functionally annotated genome map depends upon studies in model organisms, not least the mouse. Further, recent advances in genetic manipulation and in vivo, in vitro, and in silico phenotyping technologies in the mouse make annotation of the vast majority of functional elements within the mammalian genome feasible. The implementation of a Deep Genome Project—to deliver the functional biological annotation of all human orthologous genomic elements in mice—is an essential and executable strategy to transform our understanding of genetic and genomic variation in human health and disease that will catalyze delivery of the promised benefits of genomic medicine to children and adults around the world.

## Rationale

A comprehensive understanding of genetics, at the single locus, gene, and genomic level, and the pathophysiological consequences of gene variation resulting in gene, RNA, or protein dysfunction are crucial to meeting societal expectations of precision medicine and critical to optimizing clinical practice. With over 80% conserved synteny and a high degree of gene orthology, the mouse and human genomes have provided a unique opportunity for comparative functional analysis and the use of genetically altered mice to interrogate the pathobiology of human disease [1]. For example, of the 6000–8000 rare genetic diseases cited by the rare disease community, the genetic basis is known for between 5000 and 6000 [2], many of which were revealed and/or confirmed by studying the causative genetic variants in mice. Nevertheless, when viewed from a genotype perspective, more than 75 to 80% of the computationally annotated ~ 20,000 genes in the human genome have not had variation in them tied to any specific phenotype [3].

CRISPR/Cas9 has enabled rapid and highly efficient targeted mutagenesis of the mouse genome. Concurrent development of in vivo analytical and imaging technologies has transformed high-throughput pipelines for precise and reproducible phenotyping of mouse mutants [4]. Deep phenotyping of virtually all body systems including cardiovascular, digestive, endocrine, immune, integumentary, lymphatic, muscular, neurological, sensory, reproductive, respiratory, skeletal, and urinary systems is possible. Further, online computational resources such as the MONARCH Initiative (www.monarchinitiative.org) that use controlled vocabularies to integrate numeric, text, and image biological information from heterogeneous datasets (e.g., MGI, OMIM, Orphanet) link genotype to phenotype and enable comparisons between mouse and human ontologies [5]. These and other advances have facilitated the coordination of industrial scale mutant mouse production and phenotyping at costs far less than previously imagined making functional annotation of all human orthologous genomic elements in mice an achievable scientific goal.

Realization of this goal is the foundation of the work of the International Mouse Phenotyping Consortium (IMPC). The IMPC is a coordinated program of 20 research laboratories in 12 countries on 5 continents dedicated to the design, production, and description of the function of human gene orthologs in the mouse genome (www.mousephenotype.org). The magnitude of this global effort reflects the spirit and scale of the Human Genome Sequencing Project. The IMPC uses homologous recombination in embryonic stem (ES) cells and CRISPR/Cas9 technology to create mutants for genes in the mouse genome followed by whole organism phenotyping of female and male cohorts of adult mice and embryos [6]. The focus thus far has been on the production and phenotyping of null protein-coding alleles in the mouse genome [7] recognizing that such resources serve as the fundamental baseline for mammalian gene function upon which the generation and study of allelic series of other mutations—hypomorphic, neomorphic, antimorphic, and hypermorphic—will prosper and will deliver further insights into gene-phenotype relationships.

Gene association with a broad diversity of human diseases, including hearing loss, ocular diseases, metabolic disorders, bone pathologies, developmental abnormalities, and others, differentiated by sex, has been revealed through IMPC-led discovery research and IMPC-fueled studies by the broader scientific community (https://www.mousephenotype.org/data/publications). This work continues on an industrial scale generating novel insights into gene-based disease phenotypes and other scientific domains such as conservation and ecology. This combined mouse production, phenotyping, and informatics approach has recently been applied to ~ 1/3 of known Mendelian disease genes and detected significant phenotypic similarities between human disease genes and mouse knockouts (i.e., null alleles) of the orthologs for approximately half the genes [8]. At least one clinical phenotype per disease was tested for the majority (95%) of the genes and matches detected across the whole range of body systems [9].

**Fig. 1:**
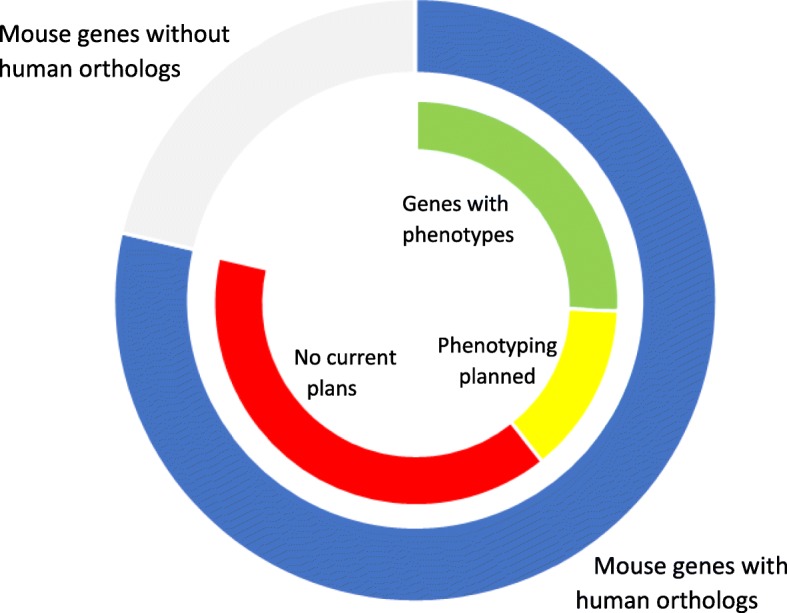
IMPC phenotyping of mouse models of human orthologous genes. *Outer ring:* Of the 22,901 genes in the mouse genome, 18,000 are human orthologs (blue) and 4901 are unique to the mouse (gray). *Inner ring:* There are currently 6255 genes (green) with phenotyping data of null alleles, and another 2925 genes (yellow) will be phenotyped over the next 2 years, leaving ~ 9000 human orthologs (red) with no plans for either production or phenotyping by the IMPC

Currently, IMPC has generated null mutations for nearly 9000 genes of which over 6000 have been phenotyped. By July 2021, IMPC will complete comprehensive phenotypic annotation for over 9000 genes, representing about half of the ~ 18,000 human orthologs in the mouse (Fig. 1). In its 10-year strategic plan for 2021–2030, the IMPC calls for expanding mouse modeling studies to inform precise molecular diagnostics and targeted therapeutics for Mendelian and multifactorial disorders to maximize beneficial impacts on human health (https://www.mousephenotypetest.org/about-impc/).

If revealing the full biological role of every gene is not daunting enough, the pathobiological effects of individual human genetic and genomic variation further escalate the challenge. As exome sequencing (ES), clinical exome sequencing (cES), and whole genome sequencing become more commonly used in research and medical diagnostics to establish an etiologic molecular diagnosis, the number of variants of unknown clinical significance (i.e., VUS) is increasing exponentially and exceeding our current capabilities to interpret loss-of-function alleles [10]. Importantly, this growth has been driven not only by clinical caregivers, but also by the growing diagnostic and perceived personal utility of these advances by other stakeholders including patients and patient families. Genetically modified mice enable statistically powered, randomized, and blinded experiments using sex-balanced and age-matched cohorts of mutant mice alongside appropriate genetic controls with sufficient sensitivity and specificity to reliably assess gene function and dysfunction in relation to specific traits, development, genetic context, and/or other physical and environmental conditions. As a result, the scale and breadth of mouse genetics research is increasingly driving the use of mouse mutants and phenotyping data to inform human genomic diagnostic projects, including the US NIH Centers for Mendelian Genomics (www.mendelian.org), the Undiagnosed Diseases Network (https://undiagnosed.hms.harvard.edu/), Canada’s Care4Rare (http://care4rare.ca), The Gabriella Miller Kids First Pediatric Research Program (https://kidsfirstdrc.org/), the Genomics England Project (https://www.genomicsengland.co.uk/), and the “Fondation Maladies Rares” (https://fondation-maladiesrares.org/eng/). Animal model data, including mice, facilitate the interpretation of potential causal variants among variants of unknown significance in clinical sequencing.

## Remaining gaps

Although substantial efforts to date have revealed a fuller understanding of the functional landscape of the entire mammalian genome, significant and important knowledge gaps remain that limit the ability to interpret the causal relationship of genes and genetic variations to human development and disease. For instance, the majority of published gene to phenotype studies continue to focus on genes that are well-annotated or for which knowledge of biological function and pathological consequences of mutations already exist [11]. As a result, much of the human genome remains unexplored and considered “dark” [12]. Strains of mouse mutants and associated phenotyping data are only available for approximately 60% of the mammalian genome, yet studies of human genes are significantly primed and enhanced by knowledge from model organisms [11]. Failure to fully illuminate the dark genome is a threat to realizing the full potential of the science of genomics, clinical genomics, the human genome project, and precision medicine. Overall, newly discovered disease genes significantly enhance the molecular diagnosis rates for clinical exome sequencing data by almost twofold [13]. Ambitious programs like the IMPC that intentionally focus on comprehensive phenotyping of genes with little to no functional annotation directly address the dark genome crisis and offer the potential for accelerated human health impact.

**Fig. 2:**
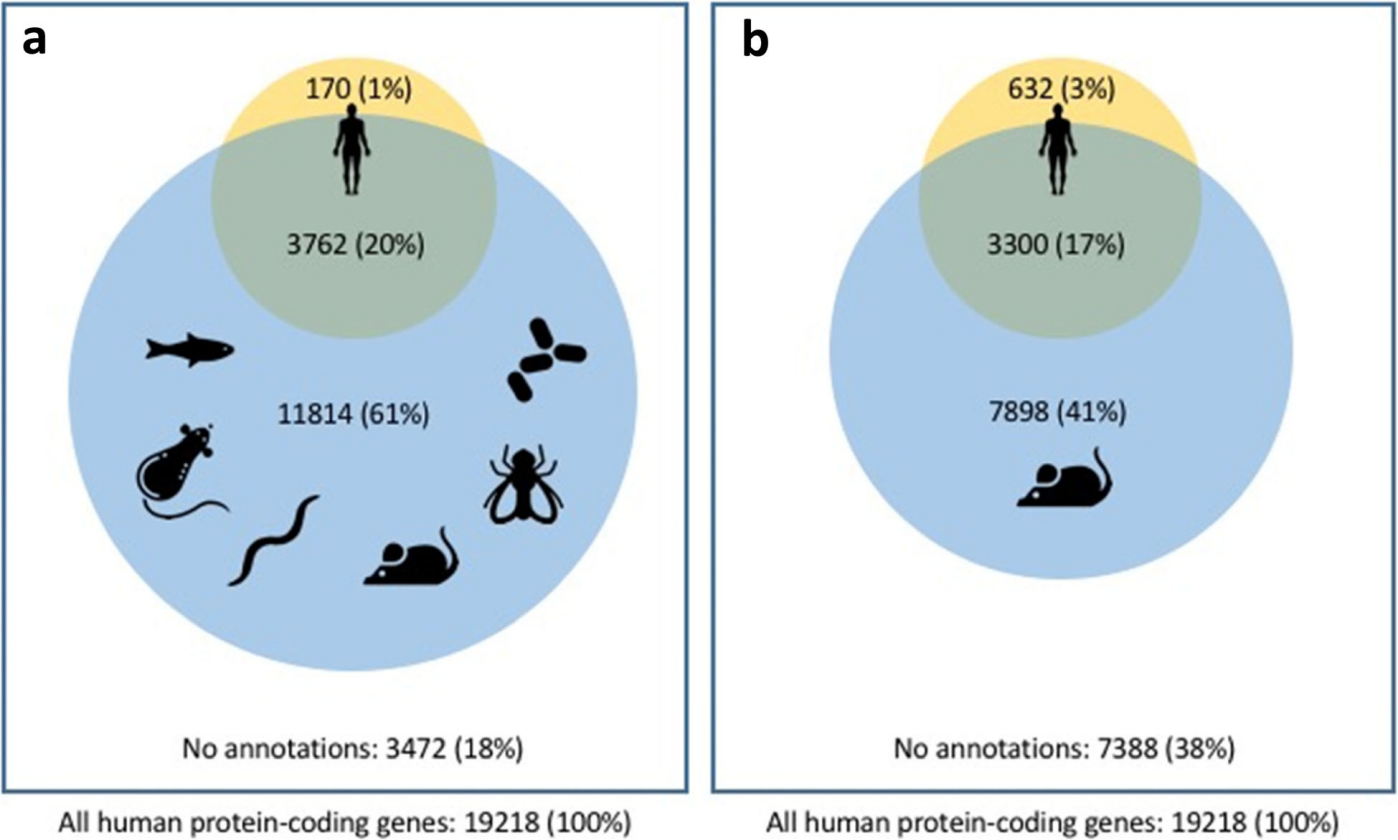
Proportion of human protein coding genes with known genotype to phenotype associations from human and **a** fish, rat, worm, mouse, fly, and yeast model organisms or **b** mouse alone. As described in the text, the depth of phenotypic coverage does not match the breadth of coverage enabled by model organisms. Human phenotypes are taken from known OMIM, Orphanet, and Clinvar Mendelian disease associations

Only around 21% of the ~ 20,000 genes annotated on the reference human genome have variation in them tied to a human disease trait (Fig. 2). Further, although human and animal (mouse, rat, fly, fish, and worm) model phenotypes together have been linked to ~ 80% of human genes, with mouse model phenotypes associated with around 60% of human genes, the depth and extent of phenotypic coverage for each gene is generally limited. Critically, for both human and model organisms, our knowledge of pleiotropy and multi-morbidities is often incomplete, undermining our understanding of gene function and disease mechanisms. Moreover, our knowledge of phenotypic heterogeneity and its potentially underlying genetic bases (e.g., locus and allelic heterogeneity, multi-locus variation, modifier loci), as well as our understanding of multiple disease phenotypes converging on a single gene locus, age-dependent penetrance, and variable expressivity, are all limited. This lack of in vivo functional annotation in experimental models contributes to long diagnostic odysseys and is a significant impediment to the development of molecular entities targeted at specific gene products [14].

The significant progress that has been made in deciphering the genetic basis of rare monogenic diseases represents the low-hanging fruit of gene to phenotype relationships. Progress in elucidating complex multi-allelic and multi-locus relationships and the consequences of de novo mutations in complex disorders is also dependent on genome-wide functional descriptions and could be further explored by the development of multi-allelic [15] and multi-locus [16] models. Mouse models have already provided significant insight into complex diseases such as juvenile diabetes [17] and autism spectrum disorders [18]. Non-mouse models have and continue to contribute immensely to this effort, but definitive identification of causal relationships between mutant alleles and human diseases will often require a mammalian model. The molecular, cellular, and physiologic insights gained from mouse studies are critical to directly inform the early recognition of predictive biomarkers before clinical symptoms manifest and to drive the identification and validation of the new therapeutic targets essential for precision medicine.

## Moving forward

We postulate that four steps undertaken by the collective endeavors of the global community will be needed to drive progress in genomic and precision medicine. These four steps, allied to genome-wide goals for in vitro systems and other model organisms, will deliver a deeper and more comprehensive understanding of individual gene function, make biological resources and data available to experimentally decipher disease mechanisms, interpret genomic variation, and reduce the diagnostic odysseys of patients with variants of unknown significance. The IMPC’s strategy 2021–2030 (https://www.mousephenotype.org/wp-content/uploads/2019/05/IMPC_Strategy_2021-30.pdf) is also formulated around these four steps.

1. *Complete functional annotation of the protein-coding genome*. Complete loss-of-function mutations are essential for identifying the phenotypic impact of protein-coding genes and a necessary first step to interpreting clinically relevant human genetic variation causing disease. By 2021, at the conclusion of the IMPC’s current mandate, ~ 9000 human orthologous genes in mice will remain to be analyzed by the consortium. Stopping at this point, halfway through the genome, would be equivalent to the Human Genome Project halting its sequencing effort after assembling euchromatic sequence of just 11 chromosomes. *It will be vital to continue efforts toward the completion of the functional analysis of the remaining unannotated protein-coding genes using mouse models.*
2. *Establish functional evaluation of the noncoding genome*. The entire coding region is only 3–5% of the mammalian genome. The remaining 95% of the genome plays many roles across a variety of biological processes, including DNA replication, transcriptional regulation, and genomic structure. Of particular note, variation in enhancers, silencers, promoters, and insulators can have significant impact on both normal and abnormal gene expression [19] and gene dosage phenomena [20]. *Strategies must be implemented for the prioritization and modeling of mutations of conserved noncoding elements in order to fully explore the in vivo function of the darkest part of the genome.*
3. *Translate functional biological knowledge to clinical knowledge*. The emerging field of genomic and precision medicine relies on the ability to interpret the potential pathophysiological consequences of genetic variation in patients. Beyond academic and research considerations, the long-term financial investments in translating human genetic variation to functional phenotypes and disease mechanisms is enormous and growing. Global investments from government agencies and corporate investors are predicted to nearly triple from US$79billion to over US$200billion in 10 years (https://bisresearch.com/industry-report/precision-medicine-market.html). For example, programs from the US All of Us Project, UK Biobank, the UK 100K Genome Project, the Chinese Precision Medicine Initiative, and many others are enrolling volunteers in efforts to gather personal history, clinical information, genome sequences, and environmental metadata to understand the role of genes in health and disease in order to identify new targets for molecular therapies. For these investments to deliver on their promise to improve health outcomes from molecular diagnosis to management and therapeutic intervention, it will be necessary to integrate gene function data generated from the study of mouse and other animal models into clinical databases, such as ClinVar, ClinGen, and others. *Continued development of model organism databases, along with improvements in data integration and analysis, is needed to enable mechanistic insight into genetic variation and disease and support future developments in genomic and precision medicine.*
4. *Enable rapid functional assessment of genomic variation and integrate functional testing into the clinical decision-making process*. While statistical inference of human patient data is currently used to discriminate disease causing from benign associated variants, a definitive molecular diagnosis is often not attainable. Even in those cases where this approach is sufficient, the delay in diagnosis is costly—psychologically, socially, and economically. *It will be necessary to undertake programs for the rapid creation and analysis of mouse models of human coding variants along with more efficient approaches to phenotyping.* These models, along with other mutational variants, will inform diagnostic decisions and targeted treatments. With these programs in place, clinicians and their research colleagues could rely on mouse models as diagnostic and therapeutic testing platforms, examine the pathological significance of a genetic variant in an orthologous mammalian system, interrogate gene/phenotype relationships for different types of alleles (e.g., SNV, etc.), explore potential gene/environment effects, and access comprehensive datasets to help guide clinical decision-making. In turn, mouse genetic experts will need to respond quickly with targeted phenotyping of mouse models in order for them to achieve clinical utility*. It will be necessary to optimize funding and bring scientific insights from mouse functional data to inform the application of mouse models as human patient avatars, make the knowledge gained from mouse data available via the electronic medical record, and enhance education and training of clinicians in genetics and genomic medicine.*

## Conclusion

Despite the incredible scientific advances in genetics at the single gene and genomic level, the collective biomedical community has only begun to scratch the surface of knowledge about the diverse and varied in vivo pathobiological roles of functional elements throughout the human genome. The global mouse genetics community is primed to address the grand challenges that we face to fully comprehend the role of genes and genetics in development, biological homeostasis, systems biology, disease, and medicine, and is ready to launch a new era for the systematic study of the function of the mammalian genome. The time is right to embark on a Deep Genome Project, on the scale of the Human Genome Project, that will fundamentally enhance the knowledgebase across the biomedical sciences. Driven by the enormous potential of mouse genetics and allied to developments in other model organisms and in vitro approaches, this project will be transformative for biology, medicine, and global health.
